# Persistent expression of chemokine and chemokine receptor RNAs at primary and latent sites of herpes simplex virus 1 infection

**DOI:** 10.1186/1743-422X-1-5

**Published:** 2004-09-23

**Authors:** W James Cook, Martha F Kramer, Russell M Walker, Timothy J Burwell, Holly A Holman, Donald M Coen, David M Knipe

**Affiliations:** 1 Millennium Pharmaceuticals Inc., Cambridge, MA 02139, USA; 2 GlycoFi, Inc., 21 Lafayette Street, Suite 200, Lebanon, NH 03766, USA; 3 Department of Biological Chemistry and Molecular Pharmacology Harvard Medical School, Boston, MA 02115, USA; 4 Department of Microbiology and Molecular Genetics, Harvard Medical School, Boston, MA 02115, USA

## Abstract

Inflammatory cytokines and infiltrating T cells are readily detected in herpes simplex virus (HSV) infected mouse cornea and trigeminal ganglia (TG) during the acute phase of infection, and certain cytokines continue to be expressed at lower levels in infected TG during the subsequent latent phase. Recent results have shown that HSV infection activates Toll-like receptor signaling. Thus, we hypothesized that chemokines may be broadly expressed at both primary sites and latent sites of HSV infection for prolonged periods of time. Real-time reverse transcriptase-polymrease chain reaction (RT-PCR) to quantify expression levels of transcripts encoding chemokines and their receptors in cornea and TG following corneal infection. RNAs encoding the inflammatory-type chemokine receptors CCR1, CCR2, CCR5, and CXCR3, which are highly expressed on activated T cells, macrophages and most immature dendritic cells (DC), and the more broadly expressed CCR7, were highly expressed and strongly induced in infected cornea and TG at 3 and 10 days postinfection (dpi). Elevated levels of these RNAs persisted in both cornea and TG during the latent phase at 30 dpi. RNAs for the broadly expressed CXCR4 receptor was induced at 30 dpi but less so at 3 and 10 dpi in both cornea and TG. Transcripts for CCR3 and CCR6, receptors that are not highly expressed on activated T cells or macrophages, also appeared to be induced during acute and latent phases; however, their very low expression levels were near the limit of our detection. RNAs encoding the CCR1 and CCR5 chemokine ligands MIP-1α, MIP-1β and RANTES, and the CCR2 ligand MCP-1 were also strongly induced and persisted in cornea and TG during the latent phase. These and other recent results argue that HSV antigens or DNA can stimulate expression of chemokines, perhaps through activation of Toll-like receptors, for long periods of time at both primary and latent sites of HSV infection. These chemokines recruit activated T cells and other immune cells, including DC, that express chemokine receptors to primary and secondary sites of infection. Prolonged activation of chemokine expression could provide mechanistic explanations for certain aspects of HSV biology and pathogenesis.

## Introduction

Acute viral infections are usually cleared from the primary site of infection by the host immune response [1], but some viruses can persist at other sites in a latent form. Herpes simplex virus (HSV), for example, causes a primary infection at a mucosal site, which is cleared within 7–10 days by the host immune response. HSV, nevertheless, enters sensory neurons and establishes a latent infection within those cells. In a mouse corneal model of HSV-1 infection, infectious virus is detected in corneal secretions and tissue for approximately 7 days [2]. Similarly, infectious virus is detected in trigeminal ganglion (TG) tissue for up to approximately 10 days [2]. Latent infection is established by 30 days postinfection (dpi) because no infectious virus can be detected in homogenates of TG tissue at that time. HSV DNA, however, is readily detected in latently infected TG for at least 150 dpi [3-5]. Viral gene expression is greatly attenuated during latent infection because the only abundant viral gene product detected is the latency-associated transcript or LAT [6]. Nevertheless, low levels of lytic transcripts can be detected in ganglia latently infected with HSV [5]. Evidence of viral protein expression is provided by the continued T cell infiltration [7,8], elevated levels of interferon γ (IFN-γ) and TNF-α transcripts and numbers of IL-6 expressing cells in the ganglia, [3,9-11]. Expression of IFN-γ and TNF-α transcripts persists in TG latently infected with HSV strains unable to replicate in neurons, indicating that neither HSV replication nor ability to reactivate are required for persistent cytokine gene expression [3]. While CD4^+ ^T cells appear to be important in immunized mice for protection against challenge virus infection [12], CD8^+ ^T cells appear to be important for establishment of latent infection in mice [7]; and CD8^+ ^T cells specific for HSV persist in TG for long periods of time [8]. Thus, there is evidence for long-term immune surveillance in the ganglion during latent infection by HSV.

Chemokines are critical for recruiting inflammatory cells to infected tissues. Chemokine specificity is due in large part to the cell-specific expression of their respective receptors (reviewed in [13-15]. Inflammatory-type receptors including CCR1, CCR2, CCR5, and CXCR3 are expressed by activated T cells, macrophages, natural killer (NK) cells, and immature (*i.e*. potent for antigen capture but not antigen presentation) dendritic cells (DC), while homostatic-type receptors including CCR7 and CXCR4 are highly expressed by resting T and B cells and mature (*i.e*., antigen-presenting) DC (Table 1). In addition, receptors including CCR2, CCR5 and CXCR3 are expressed on cells (*e.g. *Th1 cells) specific for infection-induced inflammation, while others including CCR3 and CXCR4 are on cells (*e.g.*, Th2 T cells) associated with allergic inflammation. Certain receptors are expressed by specific subsets of a given cell type. For example, CCR6 is highly expressed on Langerhans-like (CD34^+^) DC that migrate to skin, but not on monocyte-derived DC that migrate to non-skin tissues (reviewed in [14]. Acute viral infection in the mouse corneal model system is known to induce the expression of cytokines and chemokines in corneal tissue. Thomas et al. [16] observed the induction of transcripts encoding N51/KC, macrophage inflammatory protein-1 β (MIP-1β), MIP-2 and monocyte chemotactic protein 1 (MCP-1) and the cytokines IL-1, IL-6, IL-12, and TNF-α. Similarly, Tumpey *et al. *[17] showed induction of MIP-2, MIP-1α, and MCP-1 chemokines in the cornea during acute infection. Infection of mouse fibroblast cells by HSV induces expression of IL-6 [18], and infection of macrophages by HSV induces RANTES expression directly [19]. Infection of other cell types may induce expression of other cytokines and chemokines. Less is known about chemokine expression during HSV latent infection phase. Halford *et al. *[10] observed RANTES RNA expression, in addition to RNAs for IL-2, TNF-α, IFN-γ, and IL-10, during latent infection.

**Table 1:** Expression of Chemokine Receptors, Chemokines and Cytokines in Leukocyte Populations

Chemokine receptors	Cell type expression	Chemokine ligand	Proposed primary function(s)
			
	
CCR1	T cells, macrophages, immature dendritic cells (DC), natural killer cells (NK)	RANTES; MIP-1α; MCP-3, and 4; HCC-1, 2, and 4	Migration of DC to sites of inflammation Recruitment of T cells, macrophages and NK
CCR2	T cells, natural killer cells (NK), macrophages, immature DC	MCP-1, 3, and 4	Migration of effector T cells (Th1) Migration of DC progenitors to sites of inflammation
CCR3	eosinophils, basophils, T cells	eotaxin-1 and 2; RANTES; MCP-2, 3, and 4; HCC-2	Recruitment of eosinophils
CCR5	T cells (Th1, Tc1), macrophages, immature DC	RANTES; MIP-1α and 1β	Migration of effector T cells (Th1) Migration of DC to sites of inflammation Recruitment of macrophages
CCR6	immature DC (CD34+/Langerhans-like), T cells	MIP-3α	Migration of DC to skin
CCR7	T cells, B cells, mature DC	SLC, ELC	Migration of naïve T cells to lymph nodes Migration of memory T cells to lymphoid tissue
			Migration of B cells Migration of DC to lymphoid tissues
CXCR3	T cells (Th1, Tc1)	IP-10, MIG, ITAC	Migration of effector T cells (Th1)
CXCR4	T cells, macrophages, DC, B cells, others including neurons	SDF-1	Migration of effector T cells (Th2)
			Migration of B cells
			Migration of hematopoietic progenitors
Chemokines		Receptor	
			
MIP-1α	T cells, NK, macrophages, others	CCR1, CCR5	Chemoattract macrophages, T cells, NK, and others
MIP-1β	T cells, NK, macrophages, others	CCR5, CCR1 (weak)	Chemoattract macrophages, T cells, and others
RANTES	T cells, NK	CCR1, CCR5, CCR3 (weak)	Chemoattract T cells and others
MCP-1	macrophages, others	CCR2	Chemoattract macrophages, T cells, NK, and others
Eotaxin-1	epithelial cells, NK, macrophages, others	CCR3	Chemoattract eosinophils
Cytokines		Receptor	
			
IFN-γ	T cells, NK	IFN-γR	Activation of antiviral response
TNF-α	macrophages, NK, others	TNF-R	Broad activation of antiviral and inflammatory response

Recent studies have shown that HSV infection activates Toll-like signaling and chemokine synthesis [20,21]. Thus, we hypothesized that HSV infection might induce prolonged expression of a broad range of chemokines at sites of acute and latent infection. Real-time quantitative RT-PCR methods have facilitated studies of immune cell RNA expression in mouse models [22,23]. We report here the use of real-time RT-PCR to monitor RNA expression of selected chemokine receptors and their chemokine ligands during HSV infection of mouse corneal and TG tissue. Our data show that RNA encoding inflammatory-type chemokine receptors and their ligands persists in infected corneas and TG long after infectious virus can be detected, suggesting prolonged chemokine production and subsequent homing of inflammatory immune cells to these tissues. Strikingly, the data demonstrate the persistent expression of chemokines and chemokine receptor genes in the apparent absence of detectable viral productive infection transcripts in infected corneas.

## Results

### Development of TaqMan^® ^RT-PCR assays to measure viral and host gene expression during acute and latent infection

To monitor RNA expression of viral and host genes during HSV infection of mice, we developed TaqMan^® ^RT-PCR assays for the quantification of transcripts from the HSV *tk *and *ICP0 *genes and from mouse genes encoding selected chemokine receptors and their ligands. In the real-time PCR assay detailed in Materials and Methods, RNA isolated from corneal and ganglionic tissue was used for synthesis of cDNA. Primers and Taqman^® ^probes for the viral or cellular genes (Table 2) were used in real-time PCR assays to measure the concentration of cDNA for each transcript.

**Table 2:** Primer and Probe Sequences

	Forward Primer	Reverse Primer	Probe*
	
**HSV**			
tk	CGAGACAATCGCGAACATCTAC	CCCCGGCCGATATCTCA	CCACACAACACCGCCTCGACCA
ICP0	CTGCGCTGCGACACCTT	CAATTGCATCCAGGTTTTCATG	TGCATGCACCGCTTCTGCATCC
**Chemokine receptor**			
CCR1	GGGTGAACGGTTCTGGAAGTAC	CAGCCATTTTGCCAGTGGTA	ACATGCCTTTGAAACAGCTGCCGAA
CCR2	ATGAGTAACTGTGTGATTGACAAGCA	GCAGCAGTGTGTCATTCCAAGA	CTCTGTCACCTGCATGGCCTGGTCT
CCR3	ACCAGCTGTGAGCAGAGTAAACAT	CACAGCAGTGGGTGTAGGCA	CACCTCAGTCACCTGCATGGCCA
CCR5	ACTGCTGCCTAAACCCTGTCA	GTTTTCGGAAGAACACTGAGAGATAA	TCCGGAACTTCTCTCCAACAAAGGCA
CCR6	TTGGTGCAGGCCCAGAAC	GAACACGAGAACCACAGCGAT	CCAAGAGGCACAGAGCCATCCGA
CCR7	CTGCTACCTCATTATCATCCGTACCT	TGATCACCTTGATGGCCTTGT	CTCCAGGCACGCAACTTTGAGCG
CXCR3	TGTAGTTGGGCTAGCTCGAACTT	ACCTGGATATATGCTGAGCTGTCA	GCATCCTGGCAGCAAAGTTACGGG
CXCR4	CTCCAAGGGCCACCAGAA	GGCAAAGAAAGCTAGGATGAGG	CGCAAGGCCCTCAAGACGACAGTC
Chemokine			
MIP-1α	TCATCGTTGACTATTTTGAAACCAG	GCCGGTTTCTCTTAGTCAGGAA	AGCCTTTGCTCCCAGCCAGGTGTC
MIP-1β	AGGGTTCTCAGCACCAATGG	GCTGCCGGGAGGTGTAAGA	CTCTGACCCTCCCACTTCCTGCTGTTT
RANTES	CTGTCATCGCTTGCTCTAGTCCTA	CGGATGGAGATGCCGATTT	ATCCCCTACTCCCACTCCGGTCCTG
MCP-1	GCTGGGTTCAGTTTCCTTAAGC	CCTAGTCTTTAGCTGTGAGACCTTCTG	AGGCCTCGCTGCTCCACATCCA
Eotaxin-1	CCTAAGACGTGCTCTGAGGGAAT	TCCCATCTGGAACTACATGAAGC	TCAGCACCAGTCGCCCAAGGACT
Cytokine			
IFN-γ	TGAGTATTGCCAAGTTTGAGGTCA	GTGGACCACTCGGATGAGCT	CCACAGGTCCAGCGCCAAGCA
TNF-α	ACAAGGCTGCCCCGACTAC	CGCAGAGAGGAGGTTGACTT	CCTCACCCACACCGTCAGCCG

* all probes FAM-5' and 3'-TAMRA

To characterize the range over which the HSV *tk *and *ICP0 *real-time PCR assays were accurate and linear, we tested 10-fold dilutions of purified HSV genomic DNA (kind gift of Jean Pesola) starting from 5.5 × 10^4 ^copies for *tk *and *ICP0 *gene levels. The HSV *tk *and *ICP0 *primer/probe sets gave linear amplification curves over 4 logs of template concentrations until the limit of detection within the linear range was reached at 55 DNA copies for *tk *and 550 copies for *ICP0 *(not shown). At these limits of detection, the threshold cycle (CT) value, which indicated the PCR cycle at which a significant increase in amplification was first detected, was 39.2 for *tk *at 55 DNA copies and 36.5 for *ICP0 *at 550 DNA copies.

Using 2-fold dilutions of uninfected mouse TG cDNA, we observed that the primer/probe sets for host genes listed in Table 2 including GAPDH gave linear amplification curves over at least 3 and up to 7 dilutions. In all cases, CT values changed by about 1 cycle for every 2-fold change in template concentration as expected (not shown). Thus our assays matched well with previously described TaqMan^® ^assays [22-24] for linearity and sensitivity.

**Figure 1:**
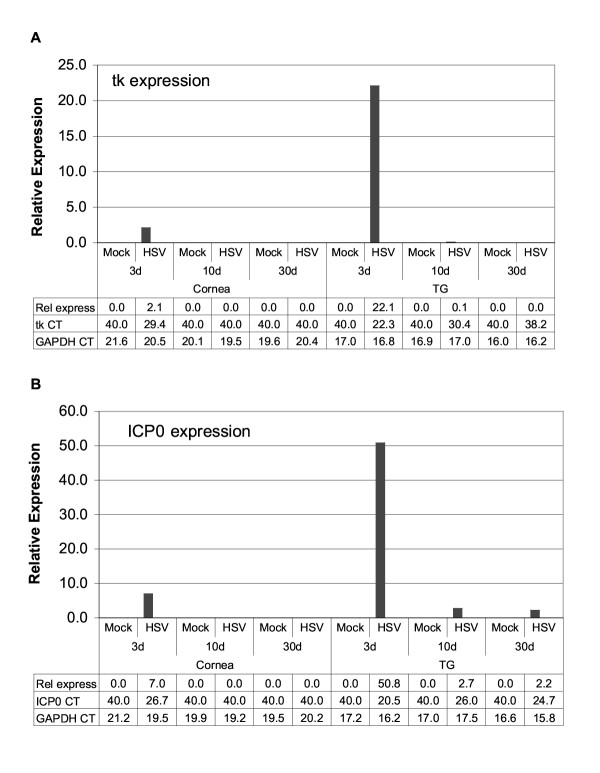
HSV *tk *and *ICP0 *RNA expression in mock and HSV-infected cornea and TG. RNA isolated from tissues harvested at 3, 10, or 30 days postinfection (d) was subjected to TaqMan RT-PCR analysis using HSV *tk *primers/probe *(A) *and *HSV *ICP0 primers/probe (B) as described in Materials and Methods. Mouse GAPDH RNA was measured in multiplex reactions, and used to calculate relative expression using the formula Rel Exp= 2^-(ΔΔCT) ^× 1000 as described in Materials and Methods. Shown below the plots are relative expression values and the CT value measured for *tk *(A) and *ICPO *(B) in each sample. The *ICP0 *signal detected at 10 and 30 dpi in HSV-infected TG is likely due to LAT RNA as described in the text. Results shown are for one experiment (Experiment #1) in which the number of individual mouse tissues pooled were 10 for cornea and 6 for TG. Similar results were obtained in two additional experiments (Experiment #2 and Experiment #3), except for variation in detection of *tk *RNA in infected TG at 30 dpi as described in the text.

Following corneal inoculation of mice with HSV or virus diluent (mock), we collected corneas and TG during acute (3 and 10 dpi) and latent (30 dpi) phases. To monitor viral gene expression in infected mice, we tested tissue samples for *tk *and *ICP0 *gene transcripts. In infected corneal tissue, HSV *tk *and *ICP0 *transcripts were readily detected at 3, but not at 10 or 30 dpi where CT values = 40 (indicating no measurable RNA) (Fig. 1). Thus we could not detect lytic transcripts in infected corneas beyond the acute phase using this assay.

In infected TG, *tk *RNA peaked at 3 dpi then dropped precipitously (200-fold) to low but readily detectable levels by 10 dpi. At 30 dpi, we detected very low or undetectable *tk *RNA expression in infected TG. In the experiment shown in Fig. 1A, we measured a CT value of 38.2 for *tk *expression in infected TG at 30 dpi, resulting in a relative expression value of 0.0002. In an independent experiment, we measured a CT of 38.1 for *tk *RNA in 30 dpi TG; however, a CT value of 40 was measured in two additional experiments (not shown). CT values for all reactions without RT were 40, indicating no DNA contamination. Thus, while *tk *expression in latent TG was at the limit of detection for our assay, our ability to detect *tk *expression in some but not all latent TG was consistent with previous reports in which very sensitive RT-PCR assays were used to detect *tk *(and *ICP0*) gene transcripts in some but not all TG during latent infection [5,25]. In those previous reports, an assay that included a radioactive Southern blotting step subsequent to RT-PCR could detect single copies of *tk *nucleic acid per PCR reaction. Our present assay for *tk *transcripts is at least 50-fold less sensitive than that used by Kramer and Coen [5].

*ICP0 *RNA levels were similar to *tk *in that they peaked at 3 dpi in cornea and TG (Fig. 1B). However, because our ICP0 probe/primer set overlaps latency-associated transcript minor (LAT) – coding sequences, the signal detected at 10 and 30 dpi in TG but not cornea may be due to minor LAT read-through RNAs. RT-PCR analysis of LAT transcripts from the TGs at 30 dpi was consistent with latent virus in infected TG (unpublished results).

### Chemokine and chemokine receptor expression in infected cornea and ganglia

We next used TaqMan^® ^RT-PCR to monitor expression of a selected series of mostly T cell and macrophage-specific chemokine receptors and chemokines in mock and HSV-infected cornea and TG. We chose chemokine receptors CCR1, CCR2, CCR5, and CXCR3, which are expressed by activated T cells, macrophages, NK cells, and immature DC that would be part of the immune infiltration in response to HSV infection, and their ligands MIP-1α, MIP-1β, RANTES, and MCP-1. For comparison, we included CCR3 which is primarily expressed on granulocytes, the CCR3 ligand eotaxin-1, CCR6 which is primarily expressed on resting T cells and immature Langerhans-like (*i.e., *skin homing) DCs, CCR7 which is primarily expressed on resting T and B cells and mature DCs that home back to lymphoid tissues, and CXCR4 which is broadly expressed on many immune and non-immune cell types (Table 1). We also tested the chemokine-inducing cytokines IFN-γ and TNF-α, whose RNA and protein have previously been shown to be expressed during both acute and latent phases of HSV infection [3,9-11].

#### i. Chemokine and chemokine receptor expression in infected cornea

**Figure 2:**
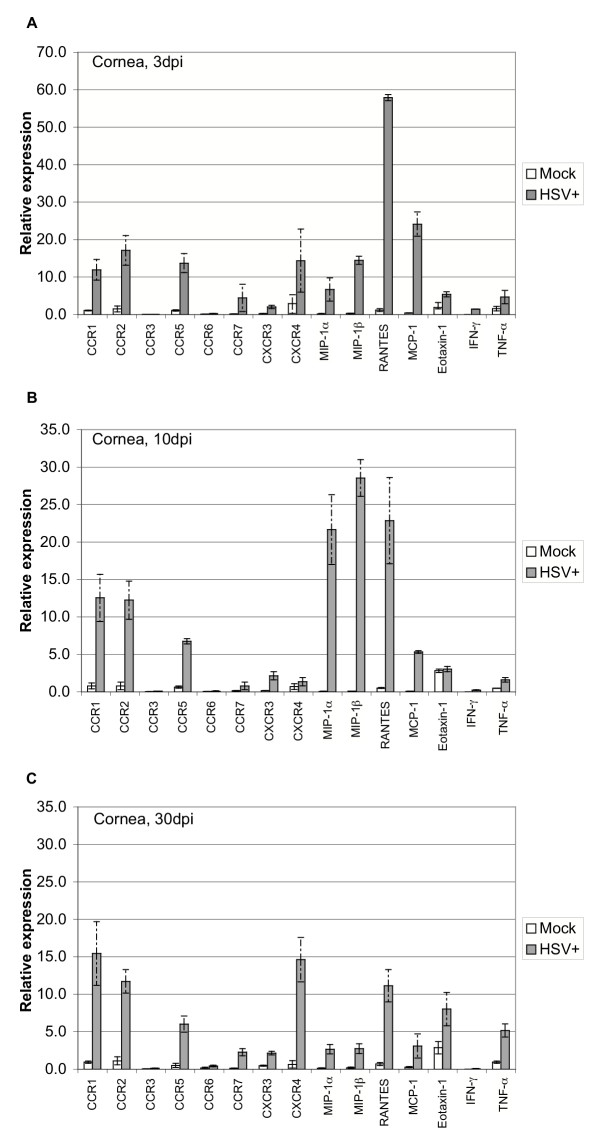
Relative levels of chemokine and chemokine receptor RNA expression in mock and HSV-infected cornea. Corneas were harvested at 3 (A), 10 (B), or 30 (C) days postinfection, and relative levels of expression were determined by TaqMan RT-PCR analysis as described in Fig. 1 and Materials and Methods. Results shown are the average of relative expression values determined using cDNA from two independent experiments, with each cDNA subjected to 2 or 3 separate measurements. Dashed bars represent ranges of individual values. Each cDNA was synthesized from RNA isolated from pooled corneas (5 mice) as described in Fig. 1 and Materials and Methods. The induction ratios (HSV+ vs. mock) for individual genes are tabulated in Table 3.

Epithelial cells of the cornea are the initial sites of replication following infection but infectious virus and viral mRNAs are not detectable past 7–10 dpi [26]. We harvested RNA from mock and HSV-infected cornea at 3, 10, and 30 dpi, and tested for chemokine receptor and chemokine RNA expression in parallel. As expected for tissues supporting active replication or having recently cleared virus, chemokine receptors CCR1, CCR2, CCR5, CCR7, CXCR3 and CXCR4, but not CCR3 or CCR6, were highly expressed and strongly induced (*i.e.*, >3-fold) at 3 and 10 dpi (Fig. 2 and Table 3). Chemokines MIP-1α, MIP-1β, RANTES, and MCP-1, but not eotaxin-1, were also highly expressed and strongly induced in infected cornea at 3 and 10 dpi. IFN-γ and TNF-α were also induced in infected cornea as previously reported [16]. Surprisingly, induction of all host RNAs tested persisted into latent phase at 30 dpi in infected corneas. For example, CCR1, CCR2, and CCR5 exhibited similar induction and similar or only slightly reduced expression levels at 30 dpi as compared to earlier time points. Relative expression and induction of CCR7 and CXCR4 in infected cornea appeared to be biphasic in that values were high at 3, lower at 10, and higher again at 30 dpi. These results suggested that continued presentation of HSV antigens stimulates chemokine production and subsequent homing of effector cells to cornea despite the apparent clearance of infectious virus.

**Table 3:** Induction Ratio (HSV+/Mock) of Transcripts for Chemokine Receptors, Chemokines and Cytokines in Cornea and Trigeminal Ganglia (TG)

	Cornea^a^			TG^b^		
		
Gene	3d	10d	30d	3d	10d	30d
					
CCR1	11 (9.2–12)	18 (13–23)	20 (10–26)	5 (2.2–7.1)	15 (9.0–19)	4 (1.7–7.0)

CCR2	14 (9.2–19)	22 (11–32)	14 (8.1–24)	3 (1.5–4.2)	15 (11–19)	3 (1.3–4.4)

CCR3	2 (1.0–5.0)	3 (2.0–5.0)	3 (2.5–3.3)	2 (0.5–5.0)	8 (2.2–20)	3 (1.8–4.7)

CCR5	12 (12.3–12.5)	11 (8.0–14)	20 (8.9–36)	9 (4.8–11)	57 (22–110)	9 (7.0–10)

CCR6	3 (2.4–3.0)	2 (1.0–2.5)	5 (1.5–8.5)	3 (0.3–11)	3 (1.0–5.0)	14 (1.0–40)

CCR7	24 (8.0–40)	5 (3.0–6.5)	17 (13–21)	13 (9.0–17)	19 (17–20)	7 (2.0–11)

CXCR3	10 (5.0–18)	15 (8.0–23)	5 (2.8–6.5)	2 (1.0–4.0)	104 (54–160)	36 (14–59)

CXCR4	11 (4.8–14)	3 (1.7–4.0)	45 (33–74)	0.6 (0.4–0.9)	4 (2.9–6.2)	3 (2.3–3.7)

MIP-1α	69 (33–106)	394 (263–1700)	34 (16–53)	232 (80–471)	126 (80–168)	25 (13–45)

MIP-1β	53 (39–67)	285 (261–310)	16 (11–21)	282 (10–595)	230 (202–245)	31 (24–37)

RANTES	55 (36–73)	43 (38–48)	16 (12–18)	64 (61–66)	304 (302–306)	31 (12–50)

MCP-1	54 (52–55)	64 (55–74)	12 (7.5–20)	153 (113–194)	22 (16–27)	3 (1.6–4.2)

Eotaxin-1	3 (1.9–3.5)	1 (0.6–1.3)	3 (1.0–5.4)	5 (3.3–9.1)	2 (1.2–2.8)	1.5 (0.7–2.3)

IFNγ	Inf.^c^	Inf.	Inf.	Inf.	Inf.	Inf.

TNF-α	3 (2.9–3.0)	3 (2.6–3.8)	7 (3.9–12)	Inf.	Inf.	Inf.

^a ^Induction ratios were calculated as relative expression in HSV-infected/relative expression in mock-infected cornea. Each value is the average of induction ratios (2 or 3 separate measurements per cDNA sample) from two independent experiments. Ranges of individual ratios are in parentheses.

^b ^Induction ratios were calculated for HSV- vs. mock-infected TG as in footnote ^a^. Each value is the average of induction ratios (2 or 3 separate measurements per cDNA sample) from three independent experiments, with ranges in parentheses.

^c ^Inf., infinite due to relative expression = 0 in all or most mock-infected samples.

#### ii. Chemokine and chemokine receptor expression in infected ganglia

**Figure 3:**
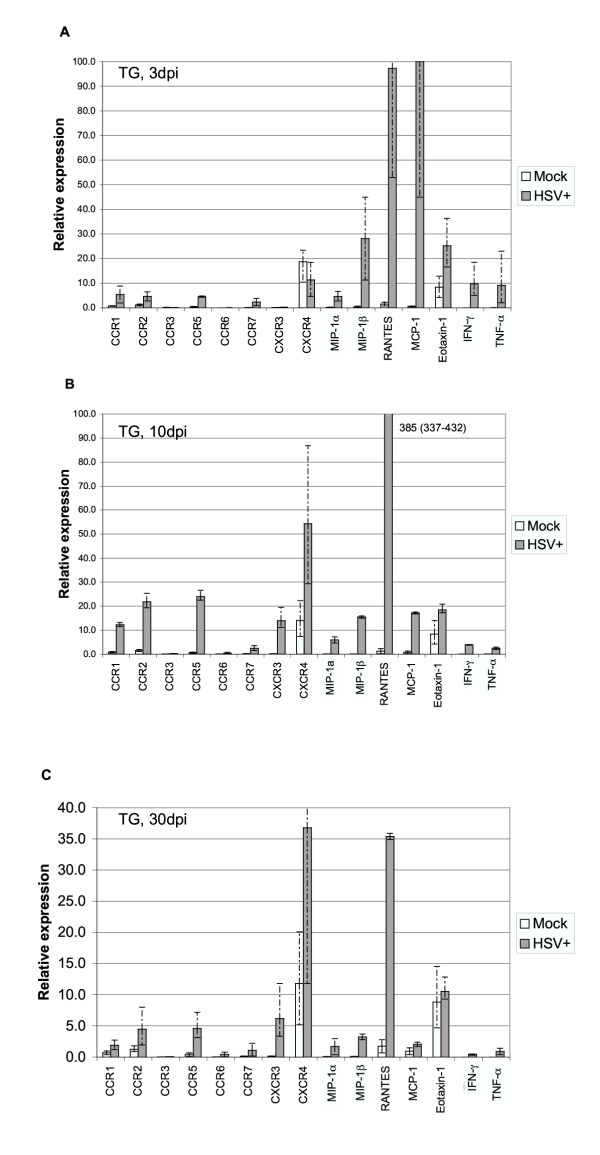
Relative levels of chemokine and chemokine receptor RNA expression in mock and HSV-infected TG. TG were harvested at 3 (A), 10 (B), or 30 (C) days postinfection, and RNA levels were determined by TaqMan RT-PCR analysis as described in Fig. 1, Fig. 2 and Materials and Methods. Results shown are the average of relative expression values determined using cDNA from three independent experiments, with each cDNA subjected to 2 or 3 separate measurements. Dashed bars represent ranges of individual values as described in Fig. 2. The induction ratios (HSV+ vs. mock) for individual genes are tabulated in Table 3.

In infected TG, transcripts from the genes encoding receptors CCR1, CCR2, CCR5, CCR7, and CXCR3 were induced by HSV infection during both acute (3 and 10 dpi) and latent (30 dpi) phases (Fig. 3 and Table 3). Peak induction of these RNAs was at 10 dpi during the clearance phase. CXCR4 was induced at 10 and 30 dpi but not at 3 dpi. While we measured induction of CCR3 and CCR6 at 10 and 30 dpi, their very low expression was at the limit of our detection (i.e., relative expression values < 0.5) as also seen in corneas. RNAs for the MIP-1α, MIP-1β, RANTES, and MCP-1 chemokines were also strongly induced at each timepoint, particularly at 3 dpi. Eotaxin-1 was induced at 3 dpi, but much less so at 10 and 30 dpi. As seen previously [3] cytokines IFN-γ and TNF-α were strongly induced at 3 and 10 dpi, but much less so at 30 dpi.

A striking finding in this analysis was the persistent expression of inflammatory cell RNAs during the latent phase of TG infection when detectable production of infectious virus has ceased. To determine if induction of these RNAs persisted past 30 dpi, we monitored expression of a limited number of transcipts from in TG collected at 45, 62, and 90 dpi. In previous studies [3-5], HSV genomic DNA was maintained at constant levels (~10^4 ^copies per TG) for up to 150 dpi in infected TG, indicating that latent virus persists well beyond 90 dpi in this mouse model. Induction of all RNAs in our panel persisted for at least 62 dpi; furthermore, all but CCR3 and eotaxin-1 were also induced at 90 dpi (Table 4). Thus chemokine receptor and ligand expression persisted long into the latent phase in infected TG.

**Table 4:** Induction Ratio (HSV+/Mock) of Transcripts for Chemokine Receptors and Chemokines in Trigeminal Ganglia (TG) at Late Times Post-Infection

	Induction Ratio^a^		
	
Gene	45d	62d	90d
			
CCR2	3 (3.2–3.3)	5 (1.3–8.8)	2 (2.2–2.4)
CCR3	8 (5.0–12)	3 (1.0–4.4)	0.7 (0.4–1.0)
CCR5	5 (5.1–5.7)	7 (4.9–9.0)	5 (2.9–6.5)
CXCR3	17 (10–24)	68 (25–111)	20 (11–28)
MIP-1α	10 (7.0–13)	35 (4.0–67)	4 (1.0–7.0)
Eotaxin-1	3 (1.5–3.9)	2 (1.1–3.1)	1.5 (0.8–2.3)

^a ^Induction ratios were calculated as relative expression in HSV-infected/relative expression in mock-infected cornea as described in Table 3. Each value is the average induction ratio (2 separate measurements per cDNA sample) from one experiment. Ranges of individual ratios are in parentheses.

## Discussion

Recent studies have shown that HSV infection induces Toll-like signaling and chemokine synthesis. Thus, we hypothesized that HSV infection might induce a broad range of chemokines at sites of primary and latent infection. In agreement with and extending previous studies [3,9-11], we have found evidence for persistent expression of chemokines and trafficking of inflammatory cells including activated T cells to acutely infected corneal tissue and to latently infected trigeminal ganglia. We also observed prolonged expression of chemokine and chemokine receptor gene transcripts in corneal tissue, the primary site of HSV-1 infection in this model system, long after infectious virus has been cleared. Microarray analysis of host gene expression has also demonstrated long-term alterations of host gene expression during latent infection by HSV, including alterations in expression of CXCR6 mRNA in TG [27]. These results argue for long-term persistence or expression of viral antigens or immunogens and stimulation of expression of these chemokines, even at the primary site of infection, the cornea. Recent results [28] have shown similar elevated chemokine expression in lung tissue after clearance of murine gamma herpesvirus 68. It will be of interest to determine how widespread this effect is among different virus infections or whether it is unique to viruses that persist in the host, such as the herpesviruses.

### Potential mechanisms for elevated expression of chemokines and chemokine receptors after viral clearance

Low level expression of viral lytic transcripts in TG during latent infection has been documented [5], which could result in low level expression of viral proteins. Recent results have shown that HSV-1 can activate Toll-like receptor 2 to stimulate chemokine expression and secretion and to activate NF-κB regulated promoters [20]. Lund *et al. *[21] showed that infectious HSV-2 and also purified HSV-2 DNA activates signaling through DC-expressed Toll-like receptor 9, resulting in the induction of IFN-α secretion. Toll-like receptor activation by HSV-2 DNA raises the intriguing possibility that HSV DNA alone is at least partially responsible for TLR-dependent induction of chemokine expression in latent TG. Among the transcripts that we studied, we detected persistent expression of transcripts for MIP-1α, MIP-1β, and RANTES, whose expression is activated by Toll-like receptors [29]. Expression of MIP-1α and MIP-1β could recruit NK cells, which express CCR5, and immature dendritic cells, which express CCR1 and CCR5, into the site of infection. Thus, elevated expression of at least some of the chemokines could be due to Toll-like receptor activation. It is also possible that other chemokines that were not assayed in this or previous studies are also induced during latent HSV infection via Toll-like receptor dependent mechanisms. Elevated expression of chemokine receptors is likely due to the chemokine-induced trafficking of inflammatory cells to the site of infection or, in the case of 30 days postinfection or latent infection, the site of viral antigen persistence.

Although we have not examined expression of IP-10, a chemokine also induced by Toll-like receptor signaling [29], we did examine the expression of transcripts for CXCR3, its receptor on activated T cells. Levels of both are elevated during latent infection in TG. Thus, stimulation of expression of this chemokine could attract activated T cells to the latently infected TG, providing a mechanism for the persistent presence of HSV-specific CD8^+ ^T cells in latently infected TG [8].

### Implications of persistent chemokine expression

Long-term inflammatory responses in neural tissue could induce pathology due to damage to neuronal cells. A number of neurological diseases have been associated with HSV infection [30], and these could be associated with these long-term inflammatory responses. In addition, the possibility of other types of specific pathological effects is raised.

#### Role of HSV in coronary heart disease

Recent data have shown an association between HSV-1 seropositivity and myocardial infarction and coronary heart disease in older adults [31]. These authors hypothesized that HSV-1 reactivation from autonomic nerves that innervate the coronary arteries could cause infection of endothelial cells, endothelial injury, and the initiation of an acute thrombotic event. Similarly, based on our work, HSV infection might induce expression of MCP-1 and IL-8, which are known to cause adhesion of monocytes to vascular endothelium [32], an early step in the development of atherosclerotic lesions in mouse models (reviewed in Gerszten et al. [32]. Therefore, the induction and prolonged expression of these chemokines by HSV infection could play a role in the pathogenesis of coronary heart disease.

#### Role of HSV in HIV transmission

Considerable evidence has accumulated for the role of genital herpes infections in promoting the transmission of human immunodeficiency virus (reviewed in [33]. Although we examined HSV-1 in these studies, HSV-2 shares many biological properties with HSV-1. Thus, it is conceivable that genital herpes infections could similarly induce the expression of chemokines in the genital mucosae and the trafficking of dendritic cells and CD4^+ ^T cells to that site. In addition to the break in the genital epithelium provided by the genital lesion, the recruitment of dendritic cells and CD4^+ ^T cells to sites of HSV infection would provide cells to transport HIV to lymph nodes and the primary host cell, respectively, and increase the potential for HIV infection.

### Implications for HSV biology and vaccine design

Recent studies on the persistence of CD8^+ ^T cells in latently infected ganglia have concluded that these cells play a role in maintaining the latent infection [8]. The results presented here raise the possibility that the presence of CD8^+ ^T cells in latently infected TG's could be the result of chemokine expression. Thus, further studies are needed to establish the causal relationship between the presence of CD8^+ ^T cells in latently infected ganglia and maintenance of latent infection.

Various HSV strains, including replication-defective mutants and amplicon vectors which do not establish neuronal latency efficiently, have been shown to induce durable immune responses [12,34,35]. These results suggest that the basis for the durable immune responses may be the persistence of antigen or continued antigen expression at sites of primary infection. Further studies are needed to determine the source of this antigen and the mechanism of the induction of chemokine expression at primary and latent sites of HSV infection.

## Materials and Methods

### Viruses, infection of mice, and tissue collection

HSV-1 KOS was propagated and titered on Vero cell monolayers as described previously [36]. Seven-week-old HSD:ICR mice (Harlan, Sprague, Dawley) were anesthetized and infected with 2 × 10^6 ^pfu of virus or mock infected with virus diluent via corneal scarification as described [2]. At specific days post infection (dpi), cornea and TG were collected and flash-frozen on dry ice with minimal elapsed time post sacrifice [5]. Cornea and TG from each time and treatment group were pooled prior to isolation of RNA. A total of four infections were performed: in Exp. #1 cornea and TG were collected at 3, 10, and 30 dpi; in Exp. #2 TG were collected at 3, 10, and 30 dpi; in Exp. #3 TG were collected at 3, 10, 45, 62, and 90 dpi; and in Exp. #4 cornea and TG were collected at 30 dpi.

### Preparation of RNA and cDNA, and real-time quantitative RT-PCR

Total RNA was purified from tissues using RNA STAT-60 (Tel-Test, Friendswood, TX), followed by secondary purification and DNAse I treatment using RNeasy columns (Qiagen). cDNA was synthesized using the Omniscript Reverse Transcriptase Kit (Qiagen) for Exp. #1 or TaqMan^® ^Reverse Transcription Reagents (Perkin Elmer) for Exps. #2, #3, and #4 following the manufacturers' suggested protocols. Design of the PCR primers and TaqMan^® ^probe**s **for mouse chemokine and chemokine receptors was done using Primer Express (Applied Biosystems) software. Primer and probe sequences are listed in Table 2. Primers and the VIC-labeled TaqMan^® ^probe**s **for the housekeeping control genes rodent GAPDH and 18S rRNA were purchased from Applied Biosystems. Real-time quantitative RT-PCR assays were performed with reagents recommended by the manufacturer (Applied Biosystems) using an ABI PRISM 7700 Sequence Detection System instrument. Briefly, 0.5 μL (approximately 300 pg) of cDNA was added to 25μL reactions containing 12.5 μL of PCR Universal Mix (Applied Biosystems), 600 nM F primer, 600 nM R primer, 200 nM FAM-labeled TaqMan probe, 200 nM rodent GAPDH F primer, 200 nM rodent GAPDH R primer, and 100 nM rodent GAPDH TaqMan^® ^probe. The number of PCR cycles needed for FAM or VIC fluorescence to cross a threshold where a statistically significant increase in change in fluorescence (CT=threshold cycle) was measured using Applied Biosystems software. Relative RNA expression was determined using the formula Rel Exp= 2^-(ΔΔCt) ^× 1000 where ΔΔ CT= (CT gene of interest-CT rodent GAPDH in experimental sample)-(CT gene of interest-CT rodent GAPDH in a no-template control sample) (the ΔΔ CT method, Taqman^® ^Bulletin #2: Relative Quantitation of Gene Expression, Applied Biosystems, updated 2001, ). To assure that GAPDH RNA levels were not affected by HSV infection and thus a good control, we repeated most analyses using 18S rRNA as an internal control. In all cases tested, induction measurements (HSV+/mock) were indistinguishable whether 18S or GAPDH were used (not shown). Control reactions lacking RT were used to test for the presence of contaminating HSV or mouse DNA, and in all cases either no or low (relative to when RT was present) levels of amplification were measured (not shown). Purified HSV-1 genomic DNA was kindly provided by Jean Pesola.

## Competing interests

The author(s) declare that they have no competing interests.

## Authors' Contributions

W. Cook, R. Walker and T. Burwell performed the RT-PCR analyses of chemokine transcripts. M. Kramer and H. Holman performed the animal infections and provided tissues for transcript analysis. D. Coen and D. Knipe participated in the design of experiments, oversight of the conduct of the experiments, and in the interpretation of the results.
